# Pelvic Actynomyces Infection: Report of Two Cases Occurred in the Hospital of San José

**DOI:** 10.1155/IDOG/2006/69020

**Published:** 2006-10-31

**Authors:** Sergio Urbina, Hernando Ruiz, Sofia Parejas

**Affiliations:** Department of Gynecology and Obstetrics, Hospital of San José, Bogotá, Colombia

## Abstract

The actynomyces infection is a rare cause of chronic pelvic inflammation, which can be manifested in multiple ways. It is caused by the actynomyces bacteria, usually by the *israelii* type, which can be a part of the normal flora of the genital tract in patients who use intrauterine device (IUD). There is a discussion about the importance of considering this infection disease as part of the differential diagnosis in patients using the IUD, with atypical manifestations and bizarre presentation of infections of the genital tract, severe pelvic adherent syndromes, tubo-ovarian complexes (abscesses) barely symptomatic, and in the case of intraoperatory suspicion of pelvic carcinomatosis among others.

*Case 1*

The patient is a 38-year old female. She was admitted to the service for a scheduled extraction of IUD by hysteroscopy. The patient was asymptomatic. She has had the IUD for 9 years. During the hysteroscopy, it was evidenced that the IUD was perforating the uterine wall at the level of the fundus. There was visualization of intestinal loops through the defect. The laparoscopic procedure was converted into a laparotomy due to the finding of sealed pelvis. The following are the findings from the exploratory laparotomy: uterine perforation, right adnexal mass in plastron, left tubo-ovarian complex perforated comprising the bowel wall at the level of the sigmoid colon, and secondary peritonitis. Hysterectomy and bilateral salpingo-oophorectomy were done. A Hartmann hemicolectomy plus drainage of the peritonitis and lavage of the peritoneal cavity were also done. The postoperative progress of the patient was adequate with absence of inflammatory response signs. The patient was discharged home. The histopathological analysis of the specimen showed infection by actynomyces comprising the uterus adnexal tissues and with severe inflammatory process of the uterine wall. Based on the results of the histopathology, the patient was readmitted to the service for intravenous antibiotic therapy. She was given crystalline penicillin 1 000 000 IU per hour by continuous infusion for 15 days. Then, she was discharged home on amoxicillin 2 grams BID, PO for six months. The patient has been followed up in two occasions one month apart in the outpatient clinic. The physical examination was within normal limits and laboratory workup showed no findings of infection or sepsis.

*Case 2*

The patient is a 23-year old female. She was admitted to the emergency room with a main complain of three-month abdominal-pelvic pain associated with the feeling of abdominal mass in some occasions, subjective fever (not assessed by thermometer) in multiple occasions, asthenia, adynamia, and weight loss. As a relevant past medical history, the patient has been diagnosed with bilateral adnexal masses, which have been followed up with tumor marker studies that are negative until this date. The past surgical history is positive for appendectomy and two c-sections. The contraception method used by the patient for the last three years was the IUD. When the patient was admitted to the service she presented a severe abdominal pain. Physical examination revealed a right adnexal mass of approximated 6 centimeters in diameter that was bounded to the body of the uterus and was not painful at manipulation. There was also a left adnexal mass of approximated 10 centimeters of diameter which was painful at palpation. Based on the findings of suffering left adnexal mass, the patient was taken to an exploratory laparotomy due to acute abdomen diagnosis. Intraoperative findings were the following: severe adhesion syndrome with sealed pelvis, and 3 centimeter diameter cystic mass in the omentum with signs of ischemia. During the surgical resection there was an incidental dissection of the intestinal serosa near the tubo-ovarian complex. Bilateral salpingo-oophorectomy was done with complete resection of the plastron and suture of the serosa was also done. Three days later the patient was readmitted by acute abdomen, she was taken back to the operation room for a new laparotomy. The findings during the laparotomy were as follows: generalized peritonitis secondary to a perforation at the level of the ileum and rectum, which required management with a colostomy. The histopathological study was compatible with bilateral tubo-ovarian abscess with infection by actynomyces. The patient received antibiotic therapy with crystalline penicillin 24 000 000 IU per day intravenously. Once the histopathology diagnosis was confirmed the patient continued therapy with ampicillin for another 6 months.

## DISCUSSION

The actynomyces infection is a chronic infection. It is caused by the actynomyces organism which is a gram positive anaerobe. The infection is usually localized around the cervicofacial area, but it could also be present with manifestations at the lungs, abdominal, or urogenital levels [1]. The infection that presents at the pelvic level corresponds to a 10 to 15% of all reported cases. There is a higher incidence of the infection in males than females, except in the abdominal and pelvic localization where the higher incidence is in females. It has been reported as an incidence of 1 to 16% of all of the pelvic infections [2]. Actynomyces is a rare cause of chronic infection; it causes local infiltration and induration of the tissues. Its role as a pathologic entity in the genital female tract is discussed because its manifestations have a wide spectrum that includes: asymptomatic infection, chronic pelvic pain, adnexal mass, tubo-ovarian complex, tumor-like carcinoma, and pelvic adhesion syndrome. The preoperative diagnosis is hard to achieve due to the finding of the microorganism by culture or immunofluorescence studies of vaginal secretion without being pathologic or any association to abdominal pain or sepsis. Although the chance of making a precise diagnosis is very attractive because it could change the therapeutic options, these facts we must consider: it is a nonmalignant process; it is a microorganism very sensible to B-lactams antibiotics; the radical surgery in some cases can be complicated with dissection of the intestinal and urinary tracts [3]. Once the diagnosis has been confirmed, the patients should be on long term therapy with B-lactams antibiotics.
